# Exposure to benzene and other hydrocarbons and risk of bladder cancer among male offshore petroleum workers

**DOI:** 10.1038/s41416-023-02357-0

**Published:** 2023-07-18

**Authors:** Nita K. Shala, Jo S. Stenehjem, Ronnie Babigumira, Fei-Chih Liu, Leon A. M. Berge, Debra T. Silverman, Melissa C. Friesen, Nathaniel Rothman, Qing Lan, H. Dean Hosgood, Sven O. Samuelsen, Magne Bråtveit, Jorunn Kirkeleit, Bettina K. Andreassen, Marit B. Veierød, Tom K. Grimsrud

**Affiliations:** 1 Department of Research, Cancer Registry of Norway, Oslo, Norway; 2 Oslo Centre for Biostatistics and Epidemiology, Department of Biostatistics, Institute of Basic Medical Sciences, University of Oslo, Oslo, Norway; 3 Division of Cancer Epidemiology and Genetics, Occupational and Environmental Epidemiology Branch, National Cancer Institute, Bethesda, MD USA; 4 Department of Epidemiology and Population Health, Albert Einstein College of Medicine, The Bronx, NY USA; 5 Department of Mathematics, University of Oslo, Oslo, Norway; 6 Department of Global Public Health and Primary Care, University of Bergen, Bergen, Norway; 7 Department of Occupational Medicine and Epidemiology, National Institute of Occupational Health, Oslo, Norway

**Keywords:** Risk factors, Cancer epidemiology, Bladder cancer

## Abstract

**Background:**

Occupational exposures constitute the second leading cause of urinary bladder cancer after tobacco smoking. Increased risks have been found in the petroleum industry, but high-quality exposure data are needed to explain these observations.

**Methods:**

Using a prospective case-cohort design, we analysed 189 bladder cancer cases (1999–2017) and 2065 randomly drawn non-cases from the Norwegian Offshore Petroleum Workers cohort. Cases were identified in the Cancer Registry of Norway, while work histories (1965–1998) and lifestyle factors were recorded by questionnaire at baseline (1998). Occupational petroleum-related hydrocarbon exposures were assessed by expert-developed job-exposure matrices. Hazard ratios were estimated by weighted Cox-regressions, adjusted for age, tobacco smoking, education, and year of first employment, and with lagged exposures.

**Results:**

Increased risks were found in benzene-exposed workers, either long-term exposure (≥18.8 years, HR = 1.89, 95% CI: 1.14–3.13; *p*-trend = 0.044) or high-level cumulative benzene exposure (HR = 1.60, 95% CI: 0.97–2.63; *p*-trend = 0.065), compared with the unexposed. Associations persisted with 20-year exposure lag. No associations were found with skin or inhalation exposure to crude oil, mineral oil (lubrication, hydraulics, turbines, drilling), or diesel exhaust.

**Conclusions:**

The results suggest that exposures in the benzene fraction of the petroleum stream may be associated with increased bladder cancer risk.

## Introduction

Pyrolysis, combustion and chemical processes based on organic compounds and fossil fuel have been linked to human cancer [1]. Cancer of the urinary bladder was recognised as an occupational disease among German industrial workers who were transforming coal tar to dyes in the second half of the 1800s [2]. In industrialised countries, tobacco smoking has since become the predominant risk factor for bladder cancer, with a population attributable risk of 50% for ever smokers [3]. Exposure to occupational carcinogens is regarded as the second leading cause, estimated to account for up to a quarter of all cases in men [4].

The International Agency for Research on Cancer (IARC) has found sufficient evidence in humans to classify 11 occupational exposures as bladder carcinogens [5], including specific agents like X- and γ-radiation, aromatic amines (i.e., 4-aminobiphenyl, benzidine, 2-naphthylamine, ortho-Toluidine), as well as some occupational activities, such as painting, firefighting, or work in the rubber or aluminium industries. Several other occupational and environmental agents are suspected of causing bladder cancer, including the solvent tetrachloroethylene, metal working fluids, diesel exhaust, polycyclic aromatic hydrocarbons (PAH) and other combustion and pyrolysis products of oil and natural gas [4–6].

An American population-based case-control study found increased risk of bladder cancer among petroleum processing workers [7], and a recent systematic review and meta-analysis of six prospective cohort studies suggested an increased risk of bladder cancer among petroleum workers in general [8]. There is a lack of specific knowledge on bladder carcinogens in the petroleum-related industry. Petroleum mainly consists of hydrocarbons, of which some compounds, such as benzene and benzo[a]pyrene, are classified by IARC as human carcinogens (Group 1). A number of PAHs, other than benzo[a]pyrene, are classified as probable or possible human carcinogens (Group 2A or B) [9, 10]. Exposure to hydrocarbons may occur by contact with the final upstream products (crude oil and natural gas) in petroleum production or elsewhere in the process (condensate and produced water), during production control and maintenance, as part of mineral oil, painting or diesel exhaust. Exposure may take place by skin contact or inhalation. Some of these exposure situations have been described earlier during the development of a job-exposure matrix for the Norwegian offshore petroleum industry [11].

In its most recent evaluation of benzene in 2017 [12], IARC reported six studies addressing mortality of bladder cancer in benzene-exposed workers, two of which also reported on incidence rates. A Nordic study based on national census data linked to a population-based benzene job-exposure matrix found a slightly increased risk of incident bladder cancer in exposed individuals compared to unexposed, although the authors concluded that concurrent effects from other solvents could not be discounted [13]. Complex, and heterogeneous exposure situations, and a lack of confounder control are common limitations in many occupational bladder cancer studies. Improved exposure assessment is needed in future studies of risk factors for bladder cancer [14].

We conducted a prospective case-cohort study to examine the risk of bladder cancer among male workers in the Norwegian offshore petroleum workers (NOPW) cohort, using industry-specific job-exposure matrices (JEM) for hydrocarbons, self-reported smoking habits, as well as high-quality incidence data by linkage to the Cancer Registry of Norway (CRN). Available JEMs included exposure to benzene, used earlier to identify expected benzene-related risks of lymphohaematopoietic cancers [15]; exposures to crude oil; mineral oil for lubrication, hydraulic and drilling purposes; and diesel exhaust.

## Material and methods

### The NOPW cohort

The NOPW cohort was established by the CRN for prospective studies of cancer incidence in the Norwegian offshore petroleum industry. The cohort has been described in detail elsewhere [16]. In short, the NOPW cohort includes 25,347 (90.8%) male and 2570 (9.2%) female offshore petroleum workers with a minimum of 20 days of work on the Norwegian continental shelf in 1965–1998, who responded to a comprehensive questionnaire in 1998 covering sociodemographic factors, work-history and lifestyle factors [16]. The response rate was estimated to 69% of the offshore workers who were invited to participate in the survey [17].

### Identification of cancer cases

The NOPW cohort was linked to the CRN for cancer diagnoses and the National Population Register for information on emigration and death by the unique 11-digit personal identification numbers, which have been assigned since 1960 to all residents in Norway. Since 1953, the CRN has registered new incident cancer diagnoses at the national level, collected from several independent sources (including pathology laboratories, physicians, the Norwegian Patient Register, and the Cause of Death Registry). This ensures a high degree of completeness and validity [18] with 94% and 98% of cancers of the urinary tract morphologically verified in 2001–2005 [19] and 2013–2017 [20], respectively.

The outcome was bladder cancer incidence (C67: International Classification of Diseases (ICD) 10th revision) during the follow-up period from 1 July 1999 to 31 December 2017. Women were excluded from the present study as only 6 bladder cancers were diagnosed among female workers, and 68% of the women worked in catering and administration with low hydrocarbon exposure [16]. Histological data was available according to ICD for Oncology 3^rd^ revision (ICD-O-3). The majority of cases (>95%) were urothelial carcinomas (ICD-O-3: 8120, 8130 and 8131), while the rest represented rare histological types (<5%), including squamous cell- and adenocarcinomas (ICD-O-3: 8070, 8255 and 8574) or unspecified (ICD-O-3: 8000).

### Study design and sample

Each worker in the NOPW cohort reported up to 8 offshore employments, which were grouped according to 5 main activities and further into 27 job categories [16]. Less than 2% reported eight employments. Information on work-history between the first and last employment had to be extracted manually, which was restricted to a representative subcohort. For the subcohort, 2268 male workers were drawn at random within strata of 5-year birth cohorts from the full NOPW cohort, according to a stratified case-cohort design [21]. A higher proportion was drawn from older birth cohorts to secure an optimum ratio of subcohort members to cases in age groups prone to develop cancer. In the NOPW cohort, 228 male workers had been diagnosed with bladder cancer before (1953–1998) or during follow-up (1999–2017).

**Fig. 1:**
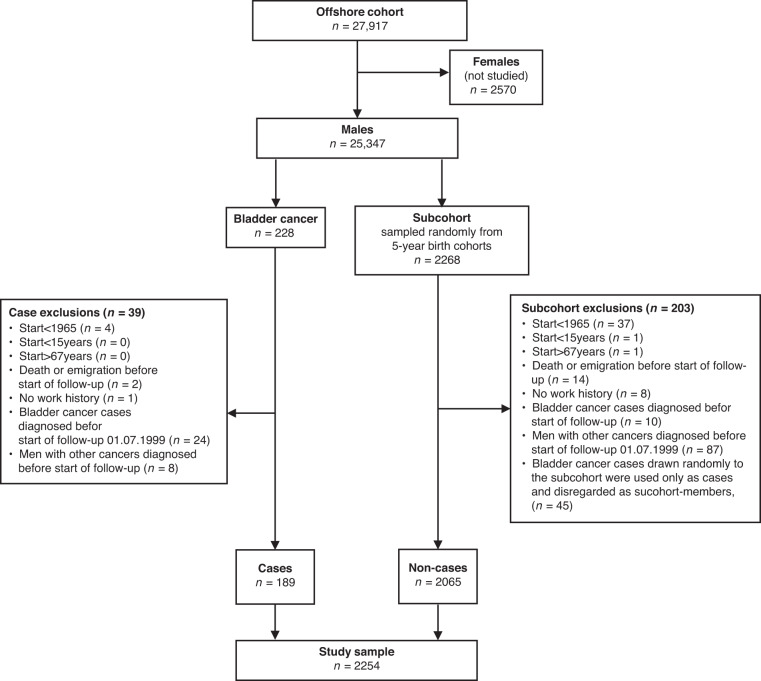
Study flowchart. Overview of the study design and sample from the Norwegian Offshore Petroleum Workers (NOPW) cohort.

We applied the following exclusion criteria on the workers with bladder cancer (*n* = 228) and the subcohort (*n* = 2268): (i) started offshore employment before 1965, (ii) age <15 or >67 years at first employment, (iii) death or emigration before start of follow-up on 1 July 1999, (iv) missing work-history, and (v) any cancer diagnosis before start of follow-up on 1 July 1999, the latter to avoid potential information and selection bias. Bladder cancer cases identified among the subcohort members were removed from the subcohort but remained in the case group as recommended for use of the ‘Borgan II estimator’ [21]. The final study sample consisted of 2254 male workers, including 189 incident bladder cancer cases and 2065 non-cases (Fig. 1).

### Assessment of exposure to hydrocarbons

#### Crude oil, mineral oil and diesel exhaust

In 2005, a group of industrial hygienists with expertise in the offshore petroleum industry working environment, developed JEMs for known or suspected carcinogenic exposures experienced in 27 pre-defined job categories in Norway’s offshore petroleum industry. Based on exposure information obtained through company visits and interviews with key personnel or compiled from exposure monitoring reports and other relevant documents, the group assessed skin exposure to crude oil or mineral oil (for lubrication or drilling fluid), and inhalatory exposure to mineral oil or diesel exhaust, during four time periods (1970–1979, 1980–1989, 1990–1999, 2000+) [11, 22]. For each combination of exposure, job category and time period, the expert group chose between the following ratings: unlikely exposed = 0; possibly exposed = 1; probably exposed = 2, i.e., at least 50% of the workers within the job category were exposed; and probably exposed = 3, for those thought to have the highest exposure intensity. Overlapping employment periods for an individual were harmonised by collapsing jobs of the same category and splitting jobs of different categories into equal periods as described in Stenehjem et al. [23]. Ever exposure was defined as a binary variable, where the unexposed workers were defined as those rated with unlikely exposure. Duration of exposure was expressed as the number of days in job categories with exposure rated >0 to the agent in question. Cumulative exposure was derived as the sum of products of exposure rating 1, 2, or 3, multiplied with duration for all employment periods in each individual work-history. For risk analyses, both duration of exposure and cumulative exposure were divided into four categories: unexposed and tertiles among the exposed cases and non-cases.

#### Exposure to benzene

In 2011, the JEM for benzene was refined following a task-oriented approach, developing semi-quantitative estimates that better captured the contrasts in intensity of benzene exposure [24]. Each job category was allowed to have several tasks involving benzene exposure. First, single tasks identified as those with a known potential for benzene exposure, were rated according to a set of 10 exposure-related determinants (concentration, temperature, personal protective equipment etc.). Second, for each job category, the benzene exposure intensity was determined as an exposure intensity score, based on the sum of products of job-category-specific duration and frequency of each relevant task, and the task-specific ratings assessed in the first step, following a procedure suggested by Hopf et al. [25]. These relative job-category-specific and time-period-specific benzene exposure intensity scores ranged from 0 to 2.4 and were linked to individual harmonised work histories. Ever exposure was defined as a binary variable, where unexposed workers only had job categories with benzene intensity score of 0. For benzene, the upper tertiles of duration of exposure and of cumulative exposure, were split by the median to capture the tail of the distribution, producing five exposure categories. Average intensity of benzene exposure was defined as the cumulative exposure divided by the total duration of exposure (in years) and was investigated as a categorical variable with an unexposed category (average intensity score = 0.0) and three categories of exposure: average intensity scores 0.05–0.5, >0.5–1.0, and >1.0 (modified from the exposure matrix in Bråtveit et al. [24] by assigning the value of .05 to categories with less certain intensity, marked with an asterisk (*) in the original paper).

### Statistical analysis

Cox proportional hazard regression, adapted to a stratified case-cohort design [21] with age as the timescale, and standard errors derived from robust variance, were used to estimate hazard ratios (HRs) and 95% confidence intervals (CIs). Test for trend across exposure categories were performed using the median values of each category. Additionally, risk was analysed according to a continuous exposure variable. Cases were assigned a weight of 1 and subcohort non-cases were assigned weights according to their inverse sampling fractions in the corresponding 5-year birth cohort stratum, thereby making the study sample representative of the full NOPW cohort. Entry time in the Cox-regressions was age at start of follow-up on 1 July 1999 and exit time age at diagnosis of bladder cancer, emigration, death or end of follow-up (31 December 2017), whichever came first.

Directed acyclic graphs (DAGs) were used to identify covariates in the multivariable models estimating the total effect of occupational hydrocarbon exposure on bladder cancer risk [26]. When the specific exposure was either crude oil, mineral oil, diesel exhaust or benzene, we used the same model (Fig. S1), called Model 1 (“the primary analysis model”) in the present study, adjusting for year of first employment (ten-year periods 1965–1998), education (compulsory, vocational, upper secondary or university/college), and tobacco smoking (never, former, current smokers < the median average intensity (daily number of cigarettes), or current smokers ≥ the median average intensity), in addition to attained age as timescale.

As a sensitivity analysis a second DAG-implied-adjustment set (Fig. S1) was investigated, applied for the benzene–bladder cancer association. In this Model 2, we adjusted for year of first employment, tobacco smoking, physical activity (never, 1–3 times/month, 1–2 times/week, 3–4 times/week or 5–7 times/week), BMI (underweight, normal weight, overweight, and obese; kg/m^2^) and work as a painter (yes or no), in addition to attained age as timescale.

In a second separate DAG (Fig. S2), we included PAH exposure as an unobserved confounding variable of the benzene–bladder cancer association. In order to adjust for potential confounding by PAH we added a summary PAH proxy variable (yes or no) to “the primary analysis model”, and named it Model 3. The DAGs and definitions of the covariates in the multivariable models are given in Supplementary file: Table S1 and Fig. S1–S2. Correlations between exposures were assessed by Spearman’s rank correlation coefficient, *r*_*s*_. And the proportional hazards assumption was evaluated by Schoenfeld residuals and found satisfactory.

Restricted cubic splines (RCS) were studied for duration of benzene exposure using five knots (0.8, 3.8, 9.5, 16.0, and 22.5 years), corresponding to the 0.05, 0.25, 0.50, 0.75 and 0.95 percentiles among exposed workers. This duration-of-exposure-risk curve was further stratified by average intensity of benzene exposure. In order to limit the influence of observations in the higher end of exposure duration, we conducted a sensitivity analysis where workers were excluded if they had >20 years of benzene exposure. A Wald test was used to test for interactions between duration of benzene exposure and average intensity of benzene exposure. Tests for interaction and the models with RCSs were based on complete-case analyses. The correlation between the two continuous variables was estimated by Pearson correlation coefficient, *r*. We also evaluated the risk of bladder cancer by duration- and cumulative benzene exposure as time-dependent exposure metrics with different exposure lag periods, disregarding exposure during the 10, 15, and 20 last years before any year of observation during follow-up.

Cancer diagnostics and therapy may influence the risk of a subsequent bladder cancer, by cause of x-radiation to the pelvic region or side-effects from chemotherapy or immune suppression [27–29]. Some bladder cancer cases may also be asymptomatic tumours detected during workup for other cancers. To avoid the impact of such activities, we performed a sensitivity analysis censoring subjects at the date of any first primary cancer diagnosis other than bladder cancer, as well as any bladder cancer diagnosed at the same date as one or more other cancers.

To evaluate a potential healthy worker survivor effect (HWSE) and facilitate a comparison with earlier studies with less information on exposures and lack of potential confounders, we investigated and reported the risks according to total employment duration and tobacco smoking in separate models. We also investigated the benzene–bladder cancer association stratified by smoking status (never and former *vs*. current), where interaction with benzene exposure was assessed with the Wald test.

Up to four percent of the study sample had one or more missing values in the tobacco smoking or education variables, or both. We used multiple imputation (MI) with chained equations to impute 20 datasets. The imputation model included all variables in the respective analysis model, as well as 5-year birth cohort, age at baseline (1998), case outcome, job category in last position, total employment duration, BMI and physical activity [30]. Each dataset was analysed separately, and the results pooled into a final point estimate with standard error using Rubin’s rule [31]. We reported results with and without imputed data. Stata version 17 was used to conduct all statistical analyses [32]. The significance level was 5%, and all tests were two-sided.

## Results

Median age at baseline was 54 for both cases and non-cases (Table 1). The maximum follow-up was 18.5 years, and less than 26% of the non-cases died or emigrated before end of follow-up. Median age at bladder cancer diagnosis for the 189 cases was 67 years (range 38–90). A total of 140 cases (74.1%) and 1434 non-cases (69.4%) were ever exposed to benzene during their work-history (Table 1). The prevalence of exposure to crude oil, mineral oil, and diesel exhaust varied between 47% and 76% of the study sample (Table 1). About four percent of the total number of yearly employment records in our study sample could not be placed in any of the 27 job categories or five main categories and were thus considered unexposed. The proportion of never smokers was lower among cases than non-cases (7% vs 22%), and the proportion of current smokers higher among cases than non-cases (48 vs 34%) (Table 1).

**Table 1:** Baseline characteristics (1998) of the study sample (*n* = 2254) in the Norwegian Offshore Petroleum Workers cohort.

Variables	Cases (*n* = 189)		Non-cases (*n* = 2065)	
Age (years), median (range)	54	(31–76)	54	(20–80)
10-year birth cohort, *n* (%)				
1915–1924	2	(1.1)	23	(1.1)
1925–1934	27	(14.3)	320	(15.5)
1935–1944	67	(35.5)	750	(36.3)
1945–1954	64	(33.9)	690	(33.4)
1955–1964	25	(13.2)	236	(11.4)
1965–1979	4	(2.1)	46	(2.2)
Education, *n* (%)				
Compulsory	29	(15.3)	327	(15.8)
Vocational	93	(49.2)	1056	(51.1)
Upper secondary	31	(16.4)	281	(13.6)
University/college	32	(16.9)	384	(18.6)
Missing	4	(2.1)	17	(0.8)
BMI (kg/m^2^), *n* (%)				
12–18.4	1	(0.5)	6	(0.3)
18.5–24.9	74	(39.2)	838	(40.6)
25.0–29.9	97	(51.3)	1012	(49.0)
≥30.0	13	(6.9)	177	(8.6)
Missing	4	(2.1)	32	(1.6)
Physical activity, *n* (%)				
Never	55	(29.1)	508	(24.6)
1–3 times/month	49	(25.9)	589	(28.5)
1–2 times/week	46	(24.3)	501	(24.3)
3–4 times/week	30	(15.9)	279	(13.5)
5–7 times/week	6	(3.2)	144	(7.0)
Missing	3	(1.6)	44	(2.1)
Tobacco smoking, *n* (%)^a^				
Never	14	(7.4)	449	(21.7)
Former	76	(40.2)	844	(40.9)
Current <12 cigarettes per day	43	(22.8)	353	(17.1)
Current ≥12 cigarettes per day	48	(25.4)	348	(16.9)
Missing	8	(4.3)	71	(3.4)
Work-history				
Year of first employment, median (range)	1978	(1965–1998)	1979	(1965–1998)
Year of first employment, *n* (%)				
1965–1974	33	(17.5)	294	(14.2)
1975–1984	103	(54.5)	1226	(59.4)
1985–1998	53	(28.0)	545	(26.4)
Total employment duration in years, median (range)	10.5	(0.3–33.0)	12.4	(0.1–33.5)
Total employment duration in years by quartiles, *n* (%)				
>0–<6.0	50	(26.5)	522	(25.3)
6.0–<12.2	54	(28.6)	502	(24.3)
12.2–<18.3	28	(14.8)	535	(25.9)
18.3–33.5	57	(30.2)	506	(24.5)
Benzene exposure				
Duration of benzene exposure (years), median (range)	5.1	(0–26.0)	4.2	(0–33.5)
Duration of benzene exposure (years), *n* (%)^b^				
0	49	(25.9)	631	(30.6)
>0–<5.5	46	(24.3)	481	(23.3)
5.5–<13.3	38	(20.1)	485	(23.5)
13.3–<18.8	23	(12.2)	239	(11.6)
18.8–33.5	33	(17.5)	229	(11.1)
Cumulative benzene exposure, median (range)	6.3	(0–43.0)	5.2	(0–51.4)
Cumulative benzene exposure, *n* (%)^b, c^				
0	49	(25.9)	631	(30.6)
>0–<2.0	47	(24.9)	479	(23.2)
2.0–<7.6	38	(20.1)	486	(23.5)
7.6–<15.3	27	(14.3)	235	(11.4)
15.3–51.4	28	(14.8)	234	(11.3)
Avg. intensity of benzene exposure, median (range)^d^	0.5	(0–1.9)	0.5	(0–1.9)
Avg. intensity of benzene exposure, *n* (%)^d^				
0	49	(25.9)	631	(30.6)
>0–0.5	78	(41.3)	823	(39.9)
>0.5–1.0	30	(15.9)	313	(15.2)
>1	32	(16.3)	298	(14.4)
Crude oil skin exposure				
Duration (years), *n* (%)^e^				
0	46	(24.3)	493	(23.9)
>0–<6.7	48	(25.4)	526	(25.5)
6.7–<14.9	45	(23.8)	525	(25.4)
14.9–33.5	50	(26.5)	521	(25.2)
Cumulative exposure, *n* (%)^c, e^				
0	46	(24.3)	493	(23.9)
>0–<7.5	42	(22.2)	532	(25.8)
7.5–<18.0	47	(24.9)	523	(25.3)
18.0–70.5	54	(28.6)	517	(25.0)
Mineral oil skin exposure				
Duration (years), *n* (%)^e^				
0	75	(39.7)	809	(39.2)
>0–<6.0	39	(20.6)	424	(20.5)
6.0–<13.5	31	(16.4)	423	(20.5)
13.5–30.7	44	(23.3)	409	(19.8)
Cumulative exposure, *n* (%)^c, e^				
0	75	(39.7)	809	(39.2)
>0–<6.7	34	(18.0)	423	(20.5)
6.7–<19.6	40	(21.2)	417	(20.2)
19.6–60.0	40	(21.2)	416	(20.2)
Mineral oil inhalation exposure				
Duration (years), *n* (%)^e^				
0	56	(29.6)	590	(28.6)
>0–<6.1	48	(25.4)	488	(23.6)
6.1–<14.0	37	(19.6)	500	(24.2)
14.0–32.0	48	(25.4)	487	(23.6)
Cumulative exposure, *n* (%)^c, e^				
0	56	(29.6)	590	(28.6)
>0–<6.8	44	(23.3)	492	(23.8)
6.8–<18.1	41	(21.7)	496	(24.0)
18.1–60.0	48	(25.4)	487	(23.6)
Diesel exhaust exposure				
Duration (years), *n* (%)^e^				
0	102	(54.0)	1087	(52.6)
>0–<4.0	30	(15.9)	325	(15.7)
4.0–<11.0	28	(14.8)	327	(15.8)
11.0–30.7	29	(15.3)	326	(15.8)
Cumulative exposure, *n* (%)^c, e^				
0	102	(54.0)	1087	(52.6)
>0–<2.4	30	(15.9)	326	(15.8)
2.4–<7.5	25	(13.2)	329	(15.9)
7.5–61.2	32	(16.9)	323	(15.6)
PAH exposure (proxy)				
Never	107	(56.6)	1226	(59.4)
Ever	82	(43.4)	839	(40.6)
Work as a painter				
Never	145	(76.7)	1606	(77.8)
Ever	44	(23.3)	459	(22.2)

^a^Tobacco-smoking status as current smoker below or above the median average intensity.

^b^Categorised into unexposed (0) and tertiles among exposed. To capture the tail of the distribution, the upper tertile was divided into two by its median.

^c^Cumulative exposure: JEM-rating multiplied by duration (in years) for each employment period and summarised for each individual from the start of employment until 31 December 1998.

^d^Average intensity: defined as the cumulative exposure divided by the total duration of exposure (in years) and investigated as a categorical variable with an unexposed group and three exposed categories.

^e^Categorised into non-exposed (0) and tertiles among exposed.

Estimates from complete-case analysis and MI analyses were similar, and in the following text we report from the MI results if not otherwise stated.

Compared to unexposed workers, a 25% increased risk of bladder cancer was suggested for workers ever exposed to benzene (HR = 1.25, 95% CI: 0.89–1.77) (Model 1, Table 2). For all exposed categories in all our benzene metrics (cumulative exposure, duration of exposure, and average intensity), and for the corresponding continuous metrics, the HRs were above unity. Compared with unexposed workers, the highest relative risk was found for those who had ≥18.8 years of exposure to benzene HR = 1.89 (95% CI: 1.14–3.13; *p*-trend = 0.044). For cumulative benzene exposure, the HR was highest for those exposed above the median of the upper exposure tertile HR = 1.60 (95% CI: 0.97–2.63; *p*-trend = 0.065), compared with unexposed workers. In Models 2 and 3 (Table 2), we included potential additional confounders (Model 2: BMI, physical activity, work as a painter; Model 3: PAH proxy variable) but the risk estimates according to benzene exposure changed negligibly. Complete-case analyses of Models 1–3 showed somewhat weaker associations but were largely similar (Table S2).

**Table 2:** Hazard ratios (HR) of bladder cancer and 95% confidence interval (95% CI) according to benzene exposure in the Norwegian Offshore Petroleum Workers cohort followed 1999–2017.

Benzene metric	Cases	Non-cases	Model 1^a^		Model 2^b^		Model 3^c^	
			HR (95% CI)^d^		HR (95% CI)^d^		HR (95% CI)^d^	
Never	49	631	1.00	(reference)	1.00	(reference)	1.00	(reference)
Ever	140	1434	1.25	(0.89–1.77)	1.28	(0.88–1.85)	1.26	(0.86–1.86)
Duration (years)^e^								
0	49	631	1.00	(reference)	1.00	(reference)	1.00	(reference)
>0–<5.5	46	481	1.18	(0.77–1.83)	1.22	(0.78–1.91)	1.18	(0.74–1.89)
5.5–<13.3	28	485	1.03	(0.66–1.60)	1.04	(0.65–1.69)	1.03	(0.63–1.67)
13.3–<18.8	23	239	1.32	(0.77–2.27)	1.34	(0.77–2.35)	1.32	(0.74–2.35)
18.8–33.5	33	229	1.89	(1.14–3.13)	1.91	(1.15–3.18)	1.87	(1.13–3.14)
*p*-trend^f^			*0.044*		*0.044*		*0.044*	
Continuous	189	2065	1.02	(1.00–1.04)	1.02	(1.00–1.04)	1.02	(1.00–1.04)
Average intensity^g^								
0	49	631	1.00	(reference)	1.00	(reference)	1.00	(reference)
>0–0.5	79	825	1.22	(0.84–1–79)	1.23	(0.83–1.83)	1.25	(0.84–1.86)
>0.5–1.0	29	312	1.11	(0.68–1.82)	1.14	(0.63–2.06)	1.18	(0.67–2.07)
>1	32	297	1.47	(0.91–2.37)	1.44	(0.89–2.34)	1.58	(0.88–2.81)
*p*-trend^f^			*0.160*		*0.181*		*0.156*	
Continuous	189	2065	1.16	(0.89–1.51)	1.15	(0.88–1.50)	1.18	(0.86–1.61)
Cumulative^e, g^								
0	49	631	1.00	(reference)	1.00	(reference)	1.00	(reference)
>0–<2.0	47	479	1.20	(0.78–1.85)	1.23	(0.80–1.92)	1.27	(0.81–2.01)
2.0–<7.6	38	486	1.05	(0.67–1.64)	1.07	(0.66–1.74)	1.11	(0.69–1.77)
7.6–<15.3	27	235	1.44	(0.86–2.41)	1.48	(0.86–2.54)	1.56	(0.91–2.67)
15.3–51.4	28	234	1.60	(0.97–2.63)	1.57	(0.94–2.62)	1.82	(1.01–3.29)
* p-*trend^f^			*0.065*		*0.084*		*0.061*	
Continuous	189	2065	1.02	(1.00–1.03)	1.02	(1.00–1.03)	1.02	(1.00–1.04)

^a^Adjusted for age (as timescale), year of first employment, tobacco smoking and education.

^b^Adjusted for age (as timescale), year of first employment, tobacco smoking, BMI and physical activity and work as a painter.

^c^Adjusted for age (as timescale), year of first employment, tobacco smoking, education and a summary PAH-proxy variable.

^d^Cox regression adapted to a case-cohort design. Missing values in covariates were imputed.

^e^Categorised into unexposed (0) and tertiles among exposed. To capture the tail of the distribution, the upper tertile was divided into two by its median.

^f^Modelled by using the medians of each category to test for linear trend.

^g^The benzene exposure metrics (average intensity and cumulative) were based on an intensity score derived from a task-based semi-quantitative JEM.

When benzene exposure was lagged, disregarding exposure occurring the last 10, 15 or 20 years before diagnosis, the positive exposure-response pattern remained largely unchanged (Table 3).

**Table 3:** Hazard ratios (HR) of bladder cancer and 95% confidence interval (95% CI) according to lagged benzene exposures in the Norwegian Offshore Petroleum Workers cohort followed 1999–2017.

	Complete-case analyses^a^				Multiple imputation analyses^a^	
	(*n* = 2156)				(*n* = 2254)	
Benzene metric	Cases	Person-years	HR (95% CI)^b^		HR (95% CI)^b^	
Duration (years)^c^						
10-year lag^d,e^						
0	47	11,101	1.00	(reference)	1.00	(reference)
>0–<5.5	46	8777	1.25	(0.81–1.92)	1.18	(0.77–1.81)
5.5–<13.3	38	8080	1.12	(0.72–1.75)	1.03	(0.66–1.60)
13.3–<18.8	24	4191	1.40	(0.82–2.40)	1.43	(0.85–2.41)
18.8–33.5	22	2671	1.61	(0.91–2.85)	1.86	(1.10–3.15)
* p*-trend^f^			*0.139*		*0.046*	
Continuous	177	34,820	1.01	(0.99–1.04)	1.02	(1.00–1.04)
15-year lag^d,e^						
0	49	12,389	1.00	(reference)	1.00	(reference)
>0–<5.5	50	9395	1.32	(0.87–1.99)	1.25	(0.83–1.86)
5.5–<13.3	44	8239	1.32	(0.86–2.02)	1.24	(0.81–1.89)
13.3–<18.8	19	3191	1.21	(0.68–2.14)	1.30	(0.76–2.24)
18.8–33.5	15	1605	1.45	(0.77–2.76)	1.64	(0.91–2.97)
* p*-trend^f^			*0.311*		*0.139*	
Continuous	177	34,820	1.01	(0.99–1.03)	1.02	(1.00–1.04)
20-year lag^d,e^						
0	57	15,076	1.00	(reference)	1.00	(reference)
>0–<5.5	52	10,351	1.28	(0.86–1.90)	1.21	(0.82–1.79)
5.5–<13.3	43	6748	1.40	(0.90–2.18)	1.34	(0.87–2.06)
13.3–<18.8	19	2050	1.57	(0.89–2.78)	1.62	(0.96–2.83)
18.8–33.5	6	594	1.26	(0.50–3.20)	1.66	(0.75–3.70)
* p*-trend^f^			*0.152*		*0.053*	
Continuous	177	34,820	1.01	(0.99–1.04)	1.02	(0.99–1.04)
Cumulative^c, g^						
10-year lag^d,e^						
0	47	11,101	1.00	(reference)	1.00	(reference)
>0–<2.0	45	8380	1.24	(0.80–1.91)	1.16	(0.76–1.78)
2.0–<7.6	39	8029	1.16	(0.74–1.81)	1.15	(0.75–1.77)
7.6–<15.3	22	3873	1.31	(0.75–2.27)	1.35	(0.80–2.28)
15.3–51.4	24	3437	1.56	(0.92–2.66)	1.59	(0.96–2.66)
* p*-trend^f^			*0.141*		*0.077*	
Continuous	177	34,820	1.01	(1.00–1.03)	1.02	(1.00–1.03)
15-year lag^d,e^						
0	49	12,389	1.00	(reference)	1.00	(reference)
>0–<2.0	48	8692	1.31	(0.86–1.99)	1.23	(0.81–1.85)
2.0–<7.6	39	7742	1.20	(0.77–1.87)	1.18	(0.77–1.82)
7.6–<15.3	18	3414	1.17	(0.65–2.10)	1.24	(0.71–2.15)
15.3–51.4	23	2583	1.79	(1.04–3.08)	1.82	(1.08–3.07)
* p*-trend^f^			*0.097*		*0.048*	
Continuous	177	34,820	1.02	(1.00–1.04)	1.02	(1.00–1.04)
20-year lag^d,e^						
0	57	15,076	1.00	(reference)	1.00	(reference)
>0–<2.0	48	8896	1.29	(0.86–1.93)	1.26	(0.84–1.87)
2.0–<7.6	37	6806	1.24	(0.79–1.95)	1.18	(0.76–1.83)
7.6–<15.3	20	2533	1.59	(0.89–2.83)	1.65	(0.95–2.84)
15.3–51.4	15	1507	1.76	(0.95–3.27)	1.74	(0.96–3.17)
* p*-trend^f^			*0.070*		*0.050*	
Continuous	177	34,820	1.02	(1.00–1.04)	1.02	(1.00–1.04)

^a^Adjusted for age as timescale, year of first employment, tobacco smoking and education (Model 1). Missing: tobacco smoking (*n* = 79), education (*n* = 21). Missing values were imputed in the multiple imputation analysis.

^b^Cox regression adapted to a case-cohort design.

^c^Time-dependent exposure.

^d^Disregarding exposure during the most recent 10-, 15-, or 20-year period before any time-point (*t*) during follow-up.

^e^Categorised into unexposed (0) and tertiles among exposed. To capture the tail of the distribution, the upper tertile was divided into two by its median.

^f^Modelled by using the medians of each exposure category to test for linear trend.

^g^The cumulative benzene exposure metric was based on an intensity score derived from a task-based semi-quantitative JEM.

**Fig. 2:**
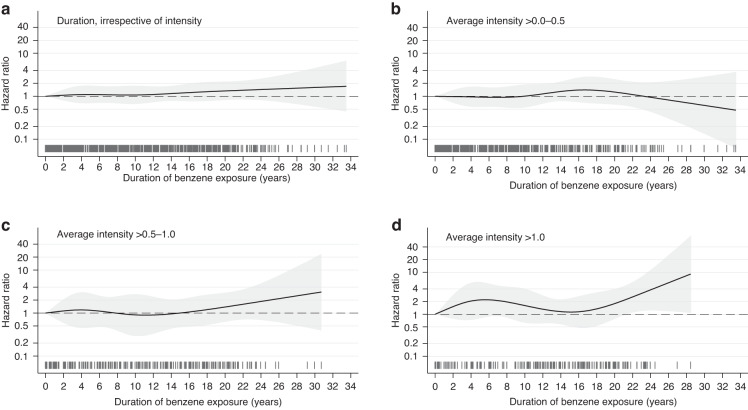
Exposure-risk curve for bladder cancer incidence according to duration of benzene exposure, and stratified by benzene average intensity. Exposure-risk curve for bladder cancer incidence and duration of benzene exposure, using a non-linear model with restricted cubic splines and five knots (0.8, 3.8, 9.5, 16.0, and 22.5 years), irrespective of average benzene intensity (**a**) and stratified by low (**b**), medium (**c**) and high (**d**) benzene average intensity. All models were adjusted for age as timescale, year of first employment, tobacco smoking and education, and based on complete-case analysis. Reference group in all models were unexposed workers. The 95% confidence interval are shown as shaded areas around the hazard ratios (solid line). The distribution of individual data points for benzene duration was illustrated by vertical lines (“rug”) along the *x*-axis”.

The restricted cubic spline (RCS) analyses of duration of benzene exposure (Fig. 2a, complete-case analyses) suggested a statistically non-significant, slight, linear increase in risk for workers with >10 years of exposure. When we stratified by benzene average intensity the lower bound of the 95% CI was >1.00 for duration over 20 years at the highest level of benzene average intensity (Fig. 2d). An elevated risk with long duration and high average intensity, persisted when workers with >20 years of benzene exposure were excluded from the study sample (Fig. S3D). However, we found no statistical interaction between duration and average intensity of benzene (*P*-interaction = 0.379). A weak positive correlation was found between benzene average intensity and duration (continuous variables, *r* = 0.194, *p* < 0.001).

We found no association between bladder cancer and skin exposure to crude oil or mineral oil, or inhalatory exposure to mineral oil or diesel exhaust, in any of the exposure metrics (Table 4). For correlations between cumulative benzene exposure and these agents (continuous variables) we found coefficients between 0.394 and 0.757 (crude oil on skin *r*_*s*_ = 0.707, *p* < 0.001; diesel exhaust *r*_*s*_ = 0.394, *p* < 0.001; mineral oil on skin *r*_*s*_ = 0.757, *p* < 0.001; and mineral oil inhalation *r*_*s*_ = 0.720, *p* < 0.001).

**Table 4:** Hazard ratios (HR) of bladder cancer and 95% confidence interval (95% CI) according to exposure to crude oil, mineral oil, and diesel exhaust in the Norwegian Offshore Petroleum Workers cohort followed 1999–2017.

	Complete-case analyse^a^				Multiple imputation analyse^a^	
	(*n* = 2156)				(*n* = 2254)	
Exposure variables	Cases	Non-cases	HR (95% CI)^b^		HR (95% CI)^b^	
Crude oil skin exposure						
Duration in years, *n* (%)^c^						
0	43	472	1.00	(reference)	1.00	(reference)
>0–<6.7	47	508	1.02	(0.65–1.59)	0.98	(0.63–1.51)
6.7–<14.9	45	501	0.95	(0.61–1.48)	0.89	(0.58–1.38)
14.9–33.5	42	498	0.87	(0.53–1.40)	0.97	(0.61–1.53)
*p*-trend^d^			*0.488*		*0.781*	
Continuous	177	1979	1.00	(0.97–1.01)	1.00	(0.98–1.02)
Cumulative exposure, *n* (%)^c,e^						
0	43	472	1.00	(reference)	1.00	(reference)
>0–<7.5	41	511	0.86	(0.54–1.37)	0.83	(0.53–1.30)
7.5–<18.0	45	500	0.95	(0.61–1.47)	0.93	(0.61–1.43)
18.0–70.5	48	496	1.02	(0.64–1,62)	1.07	(0.69–1,67)
*p*-trend^d^			*0.709*		*0.813*	
Continuous	177	1979	1.01	(0.99–1.02)	1.01	(0.99–1.02)
Mineral oil skin exposure						
Duration in years, *n* (%)^c^						
0	72	774	1.00	(reference)	1.00	(reference)
>0–<6.0	36	404	0.99	(0.64–1.53)	1.02	(0.67–1.54)
6.0–<13.5	31	404	0.83	(0.54–1.30)	0.80	(0.51–1.23)
13.5–30.7	38	397	1.07	(0.69–1.66)	1.16	(0.77–1.77)
*p*-trend^d^			*0.978*		*0.745*	
Continuous	177	1979	1.00	(0.98–1.02)	1.01	(0.99–1.03)
Cumulative exposure, *n* (%)^c,e^						
0	72	774	1.00	(reference)	1.00	(reference)
>0–<6.7	32	403	0.90	(0.57–1.41)	0.91	(0.59–1.41)
6.7–<19.6	38	397	1.06	(0.70–1.62)	1.04	(0.69–1.57)
19.6–60.0	35	405	0.92	(0.60–1.42)	1.00	(0.66–1.51)
*p*-trend^d^			*0.838*		*0.887*	
Continuous	177	1979	1.00	(0.99–1.01)	1.00	(0.99–1.02)
Mineral oil inhalation exposure						
Duration in years, *n* (%)^c^						
0	53	561	1.00	(reference)	1.00	(reference)
>0–<6.1	45	469	1.03	(0.67–1.56)	1.04	(0.69–1.57)
6.1–<14.0	37	478	0.80	(0.52–1.24)	0.78	(0.50–1.19)
14.0–32.0	42	471	0.99	(0.63–1.57)	1.09	(0.70–1.68)
*p*-trend^d^			*0.718*		*0.976*	
Continuous	177	1979	1.00	(0.97–1.02)	1.00	(0.98–1.02)
Cumulative exposure, *n* (%)^c,e^						
0	53	561	1.00	(reference)	1.00	(reference)
>0–<6.8	42	469	0.97	(0.63–1.49)	0.96	(0.63–1.46)
6.8–<18.1	39	477	0.88	(0.57–1.37)	0.90	(0.59–1.37)
18.1–60.0	43	472	0.96	(0.62–1.48)	1.03	(0.68–1.56)
*p*-trend^d^			*0.842*		*0.874*	
Continuous	177	1979	1.00	(0.99–1.01)	1.00	(0.99–1.02)
Diesel exhaust exposure						
Duration in years, *n* (%)^c^						
0	99	1049	1.00	(reference)	1.00	(reference)
>0–<4.0	28	309	0.97	(0.62–1.52)	0.98	(0.63–1.51)
4.0–<11.0	26	305	0.89	(0.56–1.41)	0.88	(0.57–1.38)
11.0–30.7	24	316	0.81	(0.50–1.29)	0.91	(0.59–1.42)
*p*-trend^d^			*0.343*		*0.623*	
Continuous	177	1979	0.99	(0.97–1.02)	1.00	(0.98–1.03)
Cumulative exposure, *n* (%)^c,e^						
0	99	1049	1.00	(reference)	1.00	(reference)
>0–<2.4	28	308	1.01	(0.64–1.58)	1.02	(0.66–1.58)
2.4–<7.5	24	311	0.82	(0.51–1.31)	0.80	(0.50–1.26)
7.5–61.2	26	311	0.84	(0.53–1.33)	0.97	(0.63–1.47)
*p*-trend^d^			*0.404*		*0.787*	
Continuous	177	1979	1.01	(0.97–1.04)	1.01	(0.98–1.04)

^a^Adjusted for age as timescale, year of first employment, tobacco smoking and education (Model 1).

^b^Cox regression adapted to a case-cohort design. Missing: tobacco smoking (*n* = 79), education (*n* = 21). Missing values were imputet in the multiple imputation analysis.

^c^Categorised into unexposed (0) and tertiles among exposed.

^d^Modelled by using the median of each category to test for linear trend.

^e^The cumulative exposure metric, for crudoil, mineral oil and diesel exhaust, was based on an exposure rating derived from a likelihood-based JEM.

Duration of total employment without information on occupational exposures, which might capture a HWSE, was not associated with bladder cancer (*p*-trend = 0.553) (Table S3). Compared to never smokers, the highest risks of bladder cancer were observed in current smokers, below and above the median average daily consumption (current smoker, <12 cigarettes/day: HR = 4.13, 95% CI: 2.21–7.70; current smoker, ≥12 cigarettes/day: HR = 5.17, 95% CI: 2.80–9.55) (Table S3).

The analysis investigating the benzene–bladder cancer association stratified by smoking status showed higher HRs and wider 95% CIs for current smokers, compared to never/former smokers (Table S4). However, no interactions were observed between benzene exposure and smoking status (0.704 ≤ *P*-interaction ≤0.732).

Bladder cancer was the first cancer diagnosed for 160 cases (median age at diagnosis 65, range 38–90); and the second, third or synchronous cancer for 29 cases (median age at diagnosis 72, range 52–86). When we restricted the outcome to bladder cancer as first primary cancer diagnosis the association with benzene was slightly strengthened for cumulative exposure and average exposure intensity (Table S5), and particularly so when an additional 20-year lag was applied to the cumulative exposure measure (relative risk for those exposed above the median of the upper exposure tertile HR = 2.16, 95% CI: 1.15–4.06, *p*-trend = 0.026) (Table S6).

## Discussion

In this prospective analysis of male offshore petroleum workers actively employed in the period 1965–1998, we sought to provide new insight into the association between exposure to petroleum-related hydrocarbons and the risk of bladder cancer. The most notable finding was the increased risk in a dose-related manner for workers with high cumulative or long-term exposure to benzene. These associations persisted when exposure was lagged 10, 15, or 20 years, when PAH exposures were adjusted for by use of a proxy summary variable, and when cases were restricted to first primary bladder cancer diagnosis. The latter approach combined with a 20-year exposure lag strengthened the evidence of an exposure-related response for the cumulative metric. We found no associations between risk of bladder cancer and exposure to crude oil, mineral oil (lubricants, hydraulics, turbines, drilling fluid) or diesel exhaust.

There was suggestion of a duration-related effect of benzene exposure at the two highest levels of average intensity when the analyses allowed for non-linear effects (RCS). The same interplay between duration and intensity was found when workers with long duration (>20 years) of benzene exposure were excluded from the analyses.

The incidence rate of bladder cancer among Norwegian offshore workers has been assessed previously with comparison to the national general population [16], and with comparison to a group of occupationally active referents [33]. In the latter study, a slight 10-percent overall elevation of the incidence of bladder cancer was suggested during the observation period 1981–2003, although not among the subgroup of upstream operators, who had a doubled risk of leukaemia and multiple myeloma likely ascribed to benzene exposure [33]. Limitations in the latter study were the lack of exposure data and a relatively low mean age of 47 years by the end of follow-up. In the present study, offshore workers were followed at older age, starting with a median age of 54 years at baseline. Age is an important risk factor for bladder cancer, illustrated by a median age at diagnosis of 73 among Norwegian men observed in parallel with the present study (1999–2017) [34].

In the most recent (1999–2017) comparison of Norwegian offshore workers with the general population [16], no evidence was found of an overall increased incidence of bladder cancer. It is interesting to note that internal comparisons (within the cohort) may point at important differences in risk, even when no overall contrast is seen compared with a large, robust, well-defined population. This observation may indicate some of the limitations in analyses that lack exposure data and confounder control.

Exposure to PAHs occurs in several industries and occupations where an elevated risk of bladder cancer has been observed [1, 35–38]. A strong dose-response relationship between bladder cancer and cumulative exposure to PAHs or benzo[a]pyrene has been found for workers at aluminium smelters [39, 40]. Upstream petroleum workers, including offshore workers involved in drilling, production, and maintenance, may also be exposed to PAHs, although not with the same degree or composition as industrial workers exposed to heated coal tar pitch volatiles in aluminium production, or in distillation of coal [41, 42]. Whether the causative agents for bladder cancer in PAH-exposed workers are PAHs themselves or aromatic amines, or other compounds accompanying these exposures has not been determined.

Our analysis was based on DAGs, and our Model 1 (“primary model”) adjusted for smoking, education, first year of employment and age as timescale. Exposure to PAHs and benzene may still occur in parallel among upstream petroleum workers and thus potentially confound our benzene risk estimates. Indeed, the correlation was quite strong (>0.7) between the cumulative benzene exposure and cumulative exposures to crude oil or mineral oil. However, we found no association with bladder cancer for crude oil and mineral oil. When our summary PAH proxy variable, which captured those having probable exposure to either of the three different agents (crude oil, mineral oil and diesel exhaust), was included in the model (Model 3), there was no evidence of confounding of the benzene–bladder cancer association. We cannot tell whether this is a sign of a true lack of PAH-related risk in the offshore environment or if residual confounding from unmeasured PAH components still exists. One might speculate that other aromatic compounds, e.g., nitro-PAHs—which may be reduced to polyaromatic amines in human cells by cytochrome P-450 enzymes [43, 44]— or other bladder carcinogens in their own right, be extracted from the petroleum stream and follow the benzene fraction.

Some aromatic amines—most often synthesised industrially from coal, tar or petroleum—are highly potent bladder carcinogens, but considered uncommon in crude oil [10]. They may exist in paint, and, as mentioned above, they have also been suggested to explain bladder cancer risk linked to production of aluminium or rubber [9, 39, 44]. Four occupational categories offshore entailed work tasks with painting and surface treatment. In a sensitivity analysis (Model 2) we adjusted for “work as a painter” and found the risks according to benzene to be largely unchanged.

In 2017, an IARC expert group evaluated six occupational cohort studies that analysed benzene-related risk of bladder cancer. IARC did not find evidence of an association with bladder cancer, but the number of cases or deaths was typically low, and data on exposure was crude or lacked information on duration [12]. A Nordic register-based case-control study on bladder cancer and occupational exposure to solvents [13], based on census data, found an exposure-related increase in risk, up to 15% above unity among men with the highest cumulative exposure to benzene (847 cases) although, as noted, it could not disentangle effects from other aromatic hydrocarbon solvents.

Benzene and many of its major metabolites are excreted in the urine [45, 46] and urinary benzene very strongly correlates with workplace air levels [47]. Further, urothelial cells possess the metabolic machinery needed to activate benzene to several of its most toxic species [48–51].

Benzene has been causally linked to myeloid leukaemia and there is some evidence for its association with lymphoid malignancies, but it has not been convincingly linked to any solid tumour to date [12]. Our current study suggests, however, the possibility of an association between benzene and bladder cancer. Although most classic chemical bladder carcinogens are potent mutagens that directly bind to DNA [4, 9] and benzene and its metabolites are considered either non-mutagenic or only weakly mutagenic [12], benzene does possess other toxicological properties that provide some biological plausibility for an association with bladder cancer [50]. Some of these DNA-damaging characteristics, especially the ability to generate a wide variety of chromosomal alterations [50], are consistent with—although not specific to—the presence of genomic changes observed in bladder cancer tumours [52].

There are also a number of carcinogens that cause both myeloid leukaemia and bladder cancer, e.g., cyclophosphamide and ionising radiation [4, 5]. Ionising radiation and benzene both generate ROS and have strong chromosomal damaging properties [12, 50]. Indeed, benzene has been referred to as a radiomimetic chemical [53]. However, cyclophosphamide, ionising radiation and even aromatic amines and schistosomiasis (recognised bladder carcinogens) have all been associated with haemorrhagic cystitis [54–56], and this may suggest a mechanism of bladder carcinogenesis that are not shared with benzene, which has not been linked to this condition.

Strengths of our study include linkage to the nationwide CRN providing complete and high-quality cancer incidence information with nearly all bladder cancers morphologically verified. The incidence rate of bladder cancer in men remained quite stable in Norway during the observation period, while bladder cancer mortality decreased about 30%. The five-year relative survival among Norwegian male bladder cancer patients is between 70 and 80%, which indicate that the statistical power is superior in an incidence study [20]. Moreover, linkage to the National Population Registry ensures control of loss to follow-up by knowledge of information on emigration and death. The recorded smoking habits seemed to capture much of the expected risk and to offer a reasonably good adjustment for potential confounding. Another strength of our study was the use of customised JEMs developed before follow-up by experts in offshore industrial hygiene for carcinogenic agents relevant for the petroleum industry. Our benzene JEM has demonstrated its ability to identify expected benzene-related risks of lymphohaematopoietic cancers in an earlier study [15]. Compared to the probabilistic JEMs for crude oil, mineral oil, and diesel exhaust, our job-task-oriented semi-quantitative benzene JEM likely resulted in less misclassification of exposure, and thus, provided a more reliable contrast between exposure levels in the job categories. All information on offshore work and potential confounding factors was collected before cancer was diagnosed, reducing the potential for differential information bias.

A limitation of our study is the lack of information on work-history during follow-up (1999–2017), which could have led to exposure misclassification and distortion of potential exposure-response associations. For lymphohaematopoietic cancers, though, the lack of recent exposure data appeared in a sensitivity analysis not to affect the risk estimates [15]. In the present study of solid tumours, as opposed to lymphohematopoietic cancers, we would expect to see a clearer dose-response when the most recent exposures were disregarded, allowing for disease latency. When risk analyses were restricted to bladder cancer as the first primary cancer, and a 20-year lag was applied, the evidence was indeed stronger for a dose-related effect from cumulative benzene exposure.

Lagging exposure has been used to address HWSE, as it reduces the opportunity for greater accrual of exposure late in the career for long-term employees, that are often healthier. However, we saw no attenuation of bladder cancer risk with increasing duration of total employment. Further, more than one-third of the cohort members terminated their offshore employment before start of follow-up, which lasted for more than 18 years. We cannot exclude the possibility that a HWSE affect our results, and a certain underestimation of the risk may therefore exist.

Work histories were self-reported, collected in a survey at baseline, but the robustness of such data has been shown by its high correlation with occupational census data and employer records [57, 58]. As the industry-specific expert-developed JEMs are retrospective, generalisations have been made when estimating exposure intensity for typical workers within the respective job categories. Thus, some misclassification exists, although the resulting bias was expected to be small or moderate [59]. However, some authors argue that differential misclassification may occur as a result of exposure categorisation, potentially biasing the estimates either towards or away from the null [60]. Some four percent of the yearly employment records were not classifiable to any of the 27 job categories or main activities, and they were thus assumed to have no exposure. Since this deficiency affected only a small proportion of the workers, we expect the bias to be negligible.

## Conclusion

Our findings suggest that high cumulative exposure and long-term exposure to benzene may be associated with increased risk of bladder cancer. Our industry-specific JEM for benzene was developed by experts in the field, using a task-based semi-quantitative approach. Still, it was not possible to determine whether the effect can be ascribed to benzene only, or to other aromatic hydrocarbons, or bladder carcinogens that may follow the benzene fraction of the petroleum stream. Exposure to benzene, PAHs and aromatic amines merit further evaluation in petroleum workers, preferably with quantitative exposure data or biological markers of exposure. Further, additional studies of occupational exposure to benzene and bladder cancer are needed to determine if the association is consistent and to explore its biological plausibility.

## Supplementary information

STROBE checklist

Supplementary Information

## Data Availability

The data are available as presented in the paper. According to Norwegian legislation, our approvals to use the data for the current study do not allow us to distribute or make the data directly available to other parties. Requests for data sharing/case pooling for projects with necessary approvals and legal basis according to the EU General Data Protection Regulation (GDPR) may be directed to the CRN; email: kreftregisteret@kreftregisteret.no.
