# Supplementary material for: Exposure to benzene and other hydrocarbons and risk of bladder cancer among male offshore petroleum workers
Source: Br J Cancer. 2023 Jul 18;129(5):838–51. doi: 10.1038/s41416-023-02357-0 (PMC10449774; doi:10.1038/s41416-023-02357-0)
Supplement: Supplementary file 2 — Supplementary Information [file 41416_2023_2357_MOESM2_ESM.docx]

**Supplementary file**

Contents

**Supplementary figures**2–4

Fig S1: Models 1 and 2 - Directed Acyclic Graph showing the relationship between hydrocarbon exposure, bladder cancer incidence, and related factors.2

Fig S2: Model 3 - Directed Acyclic Graph for the relationship between benzene exposure, bladder cancer incidence, and related factors including PAH exposure3

Fig S3: Exposure-risk curves for duration of benzene exposure, generated using restricted cubic splines: sensitivity analysis with reduced dataset4

**Supplementary tables**5–12

Table S1: Covariates and their derivation5–6

Table S2: Hazard ratios (HR) of bladder cancer and 95% confidence interval (95% CI) according to benzene exposure in the Norwegian Offshore Petroleum Workers cohort followed 1999–2017 for Models 1–3, using complete-case datasets 7

Table S3: Hazard ratios (HR) of bladder cancer and 95% confidence interval (95% CI) according to total employment duration, tobacco smoking and PAH exposure (proxy) in the Norwegian Offshore Petroleum Workers cohort followed 1999–20178

Table S4: Hazard ratios (HR) of bladder cancer and 95% confidence interval (95% CI) according to benzene exposure, full study sample and stratified by baseline smoking status, in the Norwegian Offshore Petroleum Workers cohort followed 1999–20179

Table S5: Hazard ratios (HR) of first primary bladder cancer and 95% confidence interval (95% CI) according to benzene exposure in the Norwegian Offshore Petroleum Workers cohort followed 1999–201710

Table S6: Hazard ratios (HR) of first primary bladder cancer and 95% confidence interval (95% CI) according to lagged benzene exposures in the Norwegian Offshore Petroleum Workers cohort followed 1999–201711–12

# **Supplementary Figures**


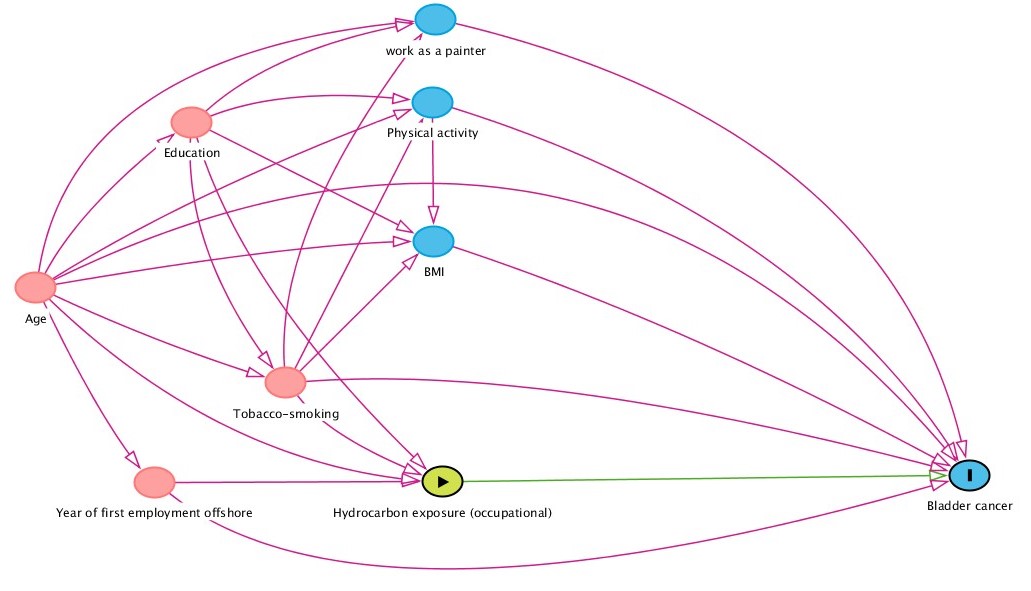


The main occupational exposure (hydrocarbon) is depicted by the green oval, and the outcome (bladder cancer) is depicted by the blue node with a black vertical line. Variables represented as pink nodes are ancestors of the exposure and outcome, the blue circles are ancestors of the outcome. The green line represents the causal path of interest, the pink lines are biasing paths. The DAG has two DAG-implied adjustment sets; Models 1 and 2. Model 1 (primary analysis) investigated a model corresponding to the adjustment set including all the pink nodes i.e., year of first employment, education, tobacco-smoking and age (as time scale). Model 2 (the second DAG-implied adjustment set, a sensitivity analysis) included year of first employment, tobacco-smoking, age (as time scale), BMI, physical activity and work as a painter (blocking the back door paths through education).

### Figure S1: Models 1 and 2 - Directed Acyclic Graph showing the relationship between hydrocarbon exposure, bladder cancer incidence, and related factors.


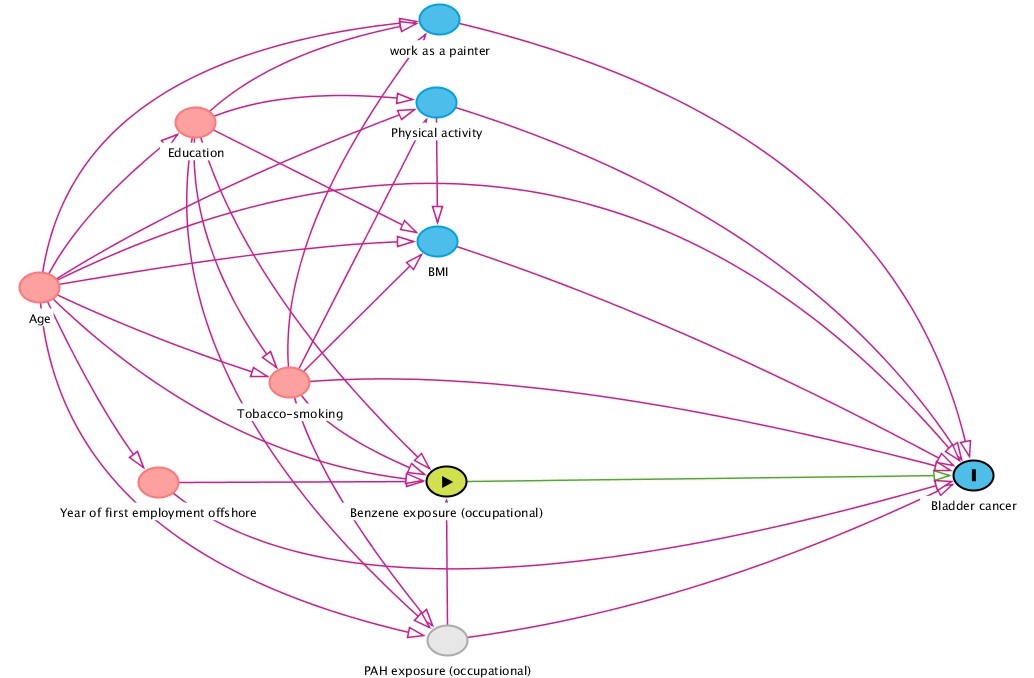


The main exposure (benzene) is depicted by the green oval, and the outcome (bladder cancer) is depicted by the blue node with a black vertical line. Variables represented as pink nodes are ancestors of the exposure and outcome, the blue circles are ancestors of the outcome, while variables represented as grey nodes are unmeasured. The green line represents the causal path of interest, and pink lines are biasing paths. In order to adjust for potential confounding by PAH we added a summary PAH proxy variable to our Model 3, also adjusting for year of first employment, education, tobacco-smoking and age (as time scale).

### Figure S2: Model 3 - Directed Acyclic Graph for the relationship between benzene exposure, bladder cancer incidence, and related factors including PAH exposure.


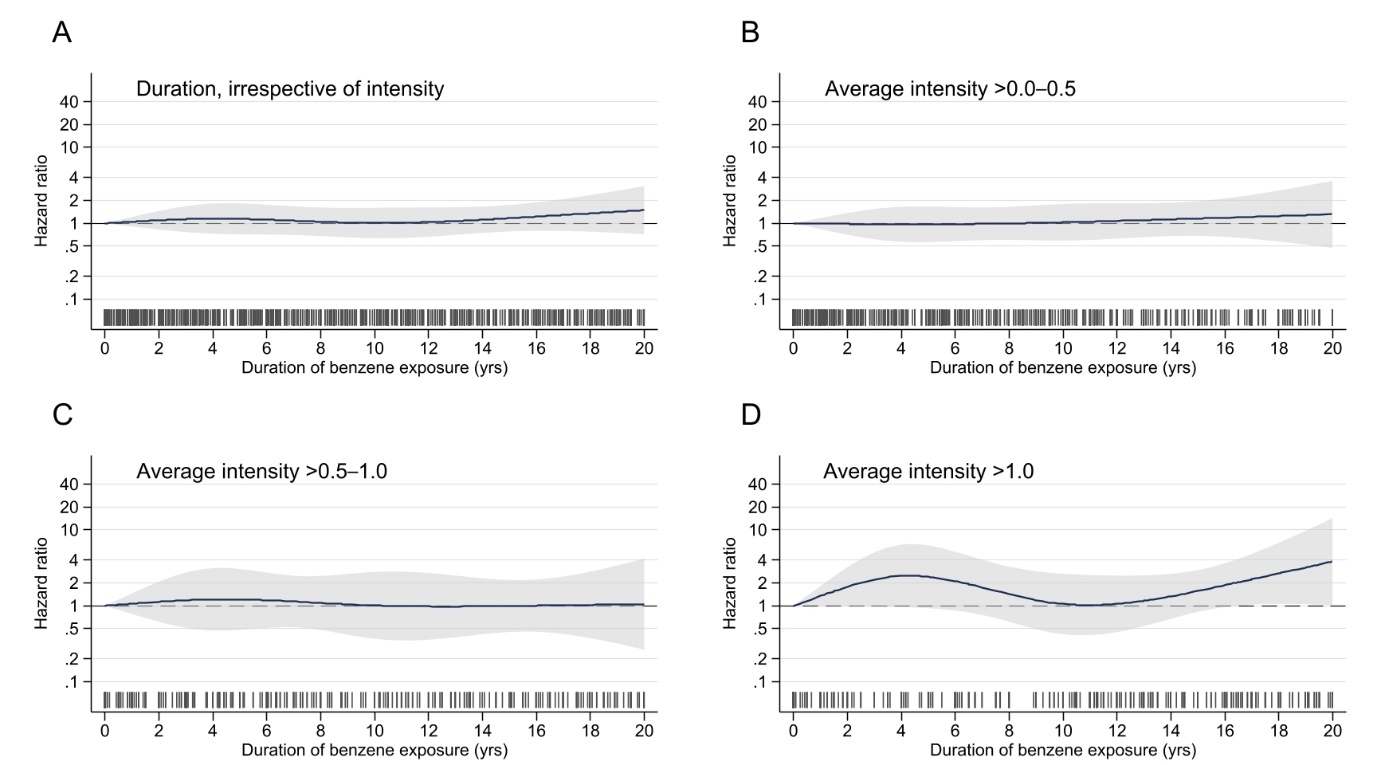


Exposure-risk curve for bladder cancer incidence and duration of benzene exposure, using a non-linear model with restricted cubic splines and four knots (0.8, 3.8, 9.5 and 16.0 years), irrespective of average benzene intensity (**A**) and stratified by low (**B**), medium (**C**) and high (**D**) benzene average intensity. All models were adjusted for age as time scale, year of first employment, tobacco smoking and education, and based on complete-case analysis. Reference group in all models were unexposed workers. The 95% confidence interval are shown as shaded areas around the hazard ratios (solid line). Workers with more than 20 years of benzene exposure were excluded in this analysis. The distribution of individual data points for benzene duration was illustrated by vertical lines (“rug”) along the x-axis”.

**Figure S3: Exposure-risk curves for duration of benzene exposure, generated using restricted cubic splines: sensitivity analysis with reduced dataset.**

# **Supplementary Tables**

### Table S1: Covariates and their derivation.

| **Covariate** | **How the variable was derived** |
| --- | --- |
| **Socio-demographic variables** | |
| Age | Information of date of birth for all workers was acquired from the National Population Register, and age was used as time scale in the analysis. |
| Education | Level of education was self-reported as either compulsory, vocational, upper secondary (folk high school and upper secondary), or university/college. |
| **Lifestyle and other variables** | |
| Tobacco smoking | Tobacco-smoking status at baseline (1998) was by each worker recorded as never, former, and current smoker, and smoking history as the number of cigarettes smoked per day in age-groups ranging from 15 to 60 + years of age. Pack-years were calculated as the sum of the daily number of cigarettes consumed (1 pack=20 cigarettes) multiplied by the number of years smoking over all age-groups. Smoking average intensity was derived by dividing pack-years by smoking duration (in years). We combined smoking status and smoking average intensity to create a 4-level smoking variable: never, former, current < the median average intensity, and current ≥ the median average intensity. |
| Body weight | Self-reported hight and weight was used to calculate body mass index (BMI, kg/m^2^). BMI was categorized according to the World Health Organization’s classification: underweight (<18.5), normal weight (18.5–24.9), overweight (25.0–29.9), and obese (≥30.0). |
| Physical activity | Each worker reported information on physical activity with an intensity at the aerobic threshold (sweaty and short of breath) for ≥20 min. Physical activity was categorized as: never, 1–3 times/month, 1–2 times/week, 3-4 times/week or 5–7 times/week. |
| **Work history variables** | |
| Year of first employment | Year of first employment was recorded as the year each worker reported starting their first employment in the offshore petroleum sector and was categorized by historical ten-year periods: 1965–1974, 1975–1984 and 1985–1998. |
| Work as painter | Work with painting and surface treatment or maintenance may have involved exposure to aromatic hydrocarbons (incl. benzene), aromatic amines, lead compounds, and epoxy resins and hardeners,^1,2^ potentially relevant for bladder cancer. The JEM report^2^ identified four job categories with probable or possible exposure as a painter (surface treatment/painters, machinists, deck crew, scaffold crew).  Work as a painter was defined as a binary variable, where exposed workers had reported employment in one or more of the identified job categories. Un-exposed worker only reported work in job categories rated as unlikely exposed. |
| PAH exposure (proxy) | A proxy variable for the un-measured variable PAH exposure, was derived using the JEMs for exposure to crude oil (skin), mineral oil (skin or inhalatory) and diesel exhaust. Exposure was defined as a binary variable, where exposed workers had reported employment in job categories rated as probably exposed either to crude oil, mineral oil or diesel exhaust. Un-exposed worker only reported work in job categories rated as unlikely or possibly exposed. |

### Table S2:

| Hazard ratios (HR) of bladder cancer and 95% confidence interval (95% CI) according to benzene exposure in the Norwegian Offshore Petroleum Workers cohort followed 1999–2017 for Models 1–3, using complete-case datasets. | | | | | | | | | |
| --- | --- | --- | --- | --- | --- | --- | --- | --- | --- |
| **Benzene metric** | **Model 1^a^** | | | **Model 2^b^** | | | **Model 3^c^** | | |
|  | **Complete-case analyses (n= 2 156)** | | | **Complete-case analyses (n= 2 123)** | | | **Complete-case analyses (n= 2 156)** | | |
|  | **Case** | **Non-case** | **HR (95% CI) ^d^** | **Case** | **Non-case** | **HR (95% CI) ^d^** | **Case** | **Non-case** | **HR (95% CI) ^d^** |
| Never | 46 | 606 | 1.00 (reference) | 47 | 594 | 1.00 (reference) | 46 | 606 | 1.00 (reference) |
| Ever | 131 | 1 373 | 1.27 (0.89, 1.81) | 131 | 1 351 | 1.26 (0.86, 1.84) | 131 | 1 373 | 1.30 (0.87, 1.93) |
|  |  |  |  |  |  |  |  |  |  |
| Duration (years) |  |  |  |  |  |  |  |  |  |
| 0 | 46 | 606 | 1.00 (reference) | 47 | 594 | 1.00 (reference) | 46 | 606 | 1.00 (reference) |
| >0–<5.5 | 45 | 461 | 1.25 (0.80, 1.94) | 45 | 450 | 1.25 (0.80, 1.97) | 45 | 461 | 1.26 (0.78, 2.04) |
| 5.5–<13.3 | 38 | 461 | 1.12 (0.72, 1.76) | 37 | 454 | 1.09 (0.67, 1.77) | 38 | 461 | 1.14 (0.69, 1.87) |
| 13.3–<18.8 | 21 | 232 | 1.27 (0.73, 2.22) | 22 | 230 | 1.30 (0.74, 2.31) | 21 | 232 | 1.29 (0.71, 2.33) |
| 18.8–33.5 | 27 | 219 | 1.69 (0.99, 2.90) | 27 | 217 | 1.65 (0.97, 2.83) | 27 | 219 | 1.71 (0.99, 2.96) |
| P-Trend ^e^ |  |  | *0.131* |  |  | *0.159* |  |  | *0.137* |
| Continuous | 177 | 1 979 | 1.01 (0.99, 1.03) | 178 | 1 945 | 1.01 (0.99, 1.03) | 177 | 1 979 | 1.01 (0.99, 1.04) |
|  |  |  |  |  |  |  |  |  |  |
| Average intensity ^f^ |  |  |  |  |  |  |  |  |  |
| 0 | 46 | 606 | 1.00 (reference) | 47 | 594 | 1.00 (reference) | 46 | 606 | 1.00 (reference) |
| >0–0.5 | 73 | 781 | 1.25 (0.85, 1.85) | 75 | 767 | 1.25 (0.83, 1.88) | 73 | 781 | 1.29 (0.86, 1.94) |
| >0.5–1.0 | 27 | 299 | 1.10 (0.66, 1.84) | 25 | 295 | 1.00 (0.53, 1.87) | 27 | 299 | 1.19 (0.66, 2.16) |
| >1 | 31 | 293 | 1.49 (0.91, 2.42) | 31 | 289 | 1.40 (0.86, 2.29) | 31 | 293 | 1.63 (0.89, 2.97) |
| P-trend ^e^ |  |  | *0.165* |  |  | *0.281* |  |  | *0.145* |
| Continuous | 177 | 1 979 | 1.17 (0.90, 1.54) | 178 | 1 945 | 1.11 (0.85, 1.47) | 177 | 1 979 | 1.21 (0.87, 1.67) |
|  |  |  |  |  |  |  |  |  |  |
| Cumulative ^f^ |  |  |  |  |  |  |  |  |  |
| 0 | 46 | 606 | 1.00 (reference) | 47 | 594 | 1.00 (reference) | 46 | 606 | 1.00 (reference) |
| >0–<2.0 | 46 | 456 | 1.28 (0.83, 1.98) | 46 | 443 | 1.29 (0.82, 2.01) | 46 | 456 | 1.36 (0.85, 2.17) |
| 2.0–<7.6 | 35 | 462 | 1.06 (0.67, 1.67) | 37 | 459 | 1.10 (0.67, 1.80) | 35 | 462 | 1.12 (0.69, 1.81) |
| 7.6–<15.3 | 24 | 228 | 1.40 (0.81, 2.40) | 23 | 224 | 1.33 (0.75, 2.36) | 24 | 228 | 1.53 (0.87, 2.70) |
| 15.3–51.4 | 26 | 227 | 1.57 (0.94, 2.64) | 25 | 225 | 1.42 (0.84, 2.43) | 26 | 227 | 1.81 (0.98, 3.37) |
| P-trend ^e^ |  |  | *0.119* |  |  | *0.273* |  |  | *0.112* |
| Continuous | 177 | 1 979 | 1.02 (1.00, 1.03) | 178 | 1 945 | 1.01 (1.00, 1.03) | 177 | 1 979 | 1.01 (1.00, 1.04) |
| **^a^** Adjusted for age (as time scale), year of first employment, tobacco smoking and education. Missing values: tobacco smoking (n=79), education (n=21). | | | | | | | | | |
| ^b^ Adjusted for age (as time scale), year of first employment, tobacco smoking, BMI and physical activity and work as a painter. Missing values: tobacco smoking (n=79), BMI (n=35) and physical activity (n=47). | | | | | | | | | |
| ^c^ Adjusted for age (as time scale), year of first employment, tobacco smoking, education and a summary PAH-proxy variable. Missing values: tobacco smoking (n=79), education (n=21). | | | | | | | | | |
| **^d^** Cox regression adapted to a case cohort design. | | | | | | | | | |
| ^e^ Modelled by using the medians of each exposure category to test for linear trend. | | | | | | | | | |
| ^f^ The benzene exposure metrics (average intensity and cumulative) were based on an intensity score derived from a task-based semi-quantitative JEM. | | | | | | | | | |

### Table S3:

| Hazard ratios (HR) of bladder cancer and 95% confidence interval (95% CI) according to total employment duration, tobacco smoking and PAH exposure in the Norwegian Offshore Petroleum Workers cohort followed 1999–2017. | | | | |
| --- | --- | --- | --- | --- |
|  | **Complete-case analyses**  **(n=2,156)** | | | **Multiple imputation analyses (n=2,254)** |
|  | **No. of cases** | **No. of**  **non-cases** | **HR (95% CI)** ^c^ | **HR (95% CI)** ^c^ |
| Total employment duration in years by quartiles ^a, b^ |  |  |  |  |
| >0–<6.0 | 48 | 504 | 1.00 (reference) | 1.00 (reference) |
| 6.0–<12.2 | 53 | 475 | 1.15 (0.76, 1.74) | 1.11 (0.74, 1.67) |
| 12.2–<18.3 | 25 | 518 | 0.49 (0.29, 0.83) | 0.56 (0.32, 0.88) |
| 18.3–33.5 | 51 | 482 | 0.97 (0.61, 1.55) | 1.01 (0.64, 1.59) |
| P-trend ^d^ |  |  | 0.420 | 0.553 |
| Continuous | 177 | 1,979 | 0.99 (0.97, 1.02) | 1.00 (0.97, 1.02) |
|  |  |  |  |  |
| Tobacco smoking ^e, b^ |  |  |  |  |
| Never | 14 | 449 | 1.00 (reference) | 1.00 (reference) |
| Former | 76 | 844 | 3.45 (1.89, 6.30) | 3.07 (1.71, 5.51) |
| Current < 12 cigarettes per day | 43 | 353 | 4.39 (2.31, 8.33) | 4.13 (2.21, 7.70) |
| Current ≥ 12 cigarettes per day | 48 | 348 | 5.38 (2.86, 10.1) | 5.17 (2.80, 9.55) |
|  |  |  |  |  |
| PAH exposure (proxy) ^a, b^ |  |  |  |  |
| Never | 101 | 1,169 | 1.00 (reference) | 1.00 (reference) |
| Ever | 76 | 810 | 1.08 (0.78, 1.48) | 1.09 (0.80, 1.48) |
|  |  |  |  |  |
| ^a^ Adjusted for age as time scale, year of first employment, tobacco smoking and education. | | | | |
| ^b^ Missing: Tobacco smoking (n=79), education (n=21). | | | | |
| ^c^ Cox regression adapted to a case cohort design. | | | | |
| ^d^ Modelled by using the median of each exposure category to test for linear trend. | | | | |
| ^e^ Adjusted for age as time scale and education | | | | |

### Table S4:

| Hazard Ratios (HR) of bladder cancer according to benzene exposure, full study sample and stratified by baseline smoking status, in the Norwegian Offshore Petroleum Workers (NOPW) cohort followed 1999–2017. | | | | | | | | | |
| --- | --- | --- | --- | --- | --- | --- | --- | --- | --- |
|  | **Full study sample ^a, b^** | | | **Smoking status at baseline** | | | | | |
|  |  |  |  | **Never and former smokers ^a, c^** | | | **Only current smokers ^a, d^** | | |
| **Benzene metric** | **Cases** | **Non-cases** | **HR (95% CI) ^e^** | **Cases** | **Non-cases** | **HR (95% CI) ^e^** | **Cases** | **Non-cases** | **HR (95% CI) ^e^** |
| Duration (years) |  |  |  |  |  |  |  |  |  |
| 0 | 48 | 611 | 1.00 (reference) | 28 | 409 | 1.00 (reference) | 20 | 202 | 1.00 (reference) |
| >0–<5.5 | 45 | 464 | 1.23 (0.80, 1.91) | 22 | 295 | 1.18 (0.64, 2.16) | 23 | 169 | 1.28 (0.67, 2.45) |
| 5.5–<13.3 | 38 | 463 | 1.07 (0.69, 1.68) | 19 | 297 | 0.98 (0.53, 1.82) | 19 | 166 | 1.21 (0.63, 2.34) |
| 13.3–<18.8 | 22 | 236 | 1.32 (0.76, 2.30) | 9 | 150 | 0.91 (0.41, 2.01) | 13 | 86 | 1.89 (0.86, 4.19) |
| 18.8–33.5 | 28 | 220 | 1.71 (1.00, 2.90) | 12 | 142 | 1.38 (0.65, 2.97) | 16 | 78 | 2.15 (0.99, 4.68) |
| *P-trend* ^f^ |  |  | *0.108* |  |  | *0.775* |  |  | *0.052* |
|  |  |  |  |  |  |  |  |  |  |
| Average intensity ^g^ |  |  |  |  |  |  |  |  |  |
| 0 | 48 | 611 | 1.00 (reference) | 28 | 409 | 1.00 (reference) | 20 | 202 | 1.00 (reference) |
| >0–0.5 | 76 | 789 | 1.27 (0.86, 1.86) | 36 | 507 | 1.11 (0.66, 1.87) | 40 | 282 | 1.44 (0.80, 2.58) |
| >0.5–1.0 | 26 | 301 | 1.07 (0.64, 1.78) | 10 | 168 | 0.92 (0.42, 2.02) | 16 | 133 | 1.24 (0.61, 2.50) |
| >1 | 31 | 293 | 1.43 (0.88, 2.30) | 16 | 209 | 1.16 (0.60, 2.23) | 15 | 84 | 1.88 (0.99, 3.89) |
| *P-trend* ^f^ |  |  | *0.236* |  |  | *0.751* |  |  | *0.142* |
|  |  |  |  |  |  |  |  |  |  |
| Cumulative ^g^ |  |  |  |  |  |  |  |  |  |
| 0 | 48 | 611 | 1.00 (reference) | 28 | 409 | 1.00 (reference) | 20 | 202 | 1.00 (reference) |
| >0–<2.0 | 46 | 458 | 1.27 (0.83, 1.96) | 22 | 275 | 1.26 (0.70, 2.28) | 24 | 183 | 1.27 (0.67, 2.40) |
| 2.0–<7.6 | 37 | 468 | 1.06 (0.68, 1.66) | 17 | 317 | 0.83 (0.43, 1.57) | 20 | 151 | 1.39 (0.72, 2.69) |
| 7.6–<15.3 | 24 | 230 | 1.37 (0.80, 2.34) | 11 | 139 | 1.23 (0.58, 2.62) | 13 | 91 | 1.53 (0.70, 3.35) |
| 15.3–51.4 | 26 | 227 | 1.54 (0.92, 2.57) | 12 | 153 | 1.24 (0.60, 2.54) | 14 | 74 | 2.05 (0.97, 4.36) |
| *P-trend* ^f^ |  |  | *0.136* |  |  | *0.635* |  |  | *0.074* |
|  | | | | | | | | | |
| ^a^ Adjusted for age (as time scale), smoking (pack-yrs. squared root transformed), education and year of first employment. | | | | | | | | | |
| ^b^ P-interaction between smoking status (never/former- vs. current smokers) and categorical variables of benzene duration, average intensity, and cumulative exposure, was 0.732, 0.710 and 0.704, respectively, assessed by the Wald test. The interaction term was not included for the model for the full study sample. | | | | | | | | | |
| ^c^ Restricted to never and former smokers at baseline 1998. | | | | | | | | | |
| ^d^ Restricted to only current smokers at baseline 1998. | | | | | | | | | |
| **^e^** Cox regression adapted to a case-cohort design. Missing values for education=19 were imputed, 79 obs. with missing in smoking history were deleted from the dataset. | | | | | | | | | |
| ^f^ Modelled by using the median of each exposure category to test for linear trend. | | | | | | | | | |
| ^g^ The benzene exposure metrics (average intensity and cumulative) were based on an intensity score derived from a task-based semi-quantitative JEM. | | | | | | | | | |

### Table S5:

| Hazard ratios (HR) of first primary bladder cancer and 95% confidence interval (95% CI) according to benzene exposure in the Norwegian Offshore Petroleum Workers cohort followed 1999–2017. | | | | |
| --- | --- | --- | --- | --- |
|  | **Complete-case analysis** ^a^  **(n=2,137)** | | | **Multiple imputation analysis** ^a^  **(n=2,231)** |
| **Benzene metric** | **No. of cases** | **No. of**  **non-cases** | **HR (95% CI)** ^b^ | **HR (95% CI)** ^b^ |
| Never | 37 | 607 | 1.00 (reference) | 1.00 (reference) |
| Ever | 116 | 1,377 | 1.40 (0.95, 2.05) | 1.37 (0.94, 1.99) |
|  |  |  |  |  |
| Duration (years)  ^d^ |  |  |  |  |
| 0 | 37 | 607 | 1.00 (reference) | 1.00 (reference) |
| >0–<5.5 | 40 | 459 | 1.36 (0.84, 2.18) | 1.31 (0.82, 2.09) |
| 5.5–<13.3 | 36 | 460 | 1.31 (0.81, 2.12) | 1.22 (0.76, 1.96) |
| 13.3–<18.8 | 17 | 238 | 1.23 (0.66, 2.29) | 1.25 (0.69, 2.29) |
| 18.8–33.5 | 23 | 220 | 1.93 (1.08, 3.45) | 2.03 (1.16, 3.54) |
| *P-trend ^e^* |  |  | *0.096* | *0.060* |
| Continuous | 153 | 1,984 | 1.02 (0.99, 1.04) | 1.02 (1.00, 1.04) |
|  |  |  |  |  |
| Average intensity ^f^ |  |  |  |  |
| 0 | 37 | 607 | 1.00 (reference) | 1.00 (reference) |
| >0–0.5 | 64 | 784 | 1.35 (0.89, 2.06) | 1.32 (0.87, 1.99) |
| >0.5–1.0 | 23 | 300 | 1.18 (0.67, 2.06) | 1.14 (0.66, 1.98) |
| >1 | 29 | 293 | 1.75 (1.04, 2.94) | 1.75 (1.05, 2.90) |
| *P-trend ^e^* |  |  | *0.062* | *0.055* |
| Continuous | 153 | 1,984 | 1.28 (0.96, 1.70) | 1.26 (0.95, 1.68) |
|  |  |  |  |  |
| Cumulative  ^d, f^ |  |  |  |  |
| 0 | 37 | 607 | 1.00 (reference) | 1.00 (reference) |
| >0–<2.0 | 41 | 455 | 1.38 (0.87, 2.20) | 1.33 (0.84, 2.10) |
| 2.0–<7.6 | 31 | 463 | 1.18 (0.72, 1.95) | 1.18 (0.73, 1.90) |
| 7.6–<15.3 | 20 | 231 | 1.47 (0.81, 2.66) | 1.44 (0.80, 2.57) |
| 15.3–51.4 | 24 | 228 | 1.79 (1.03, 3.12) | 1.80 (1.05, 3.09) |
| *P-trend ^e^* |  |  | *0.074* | *0.057* |
| Continuous | 153 | 1,984 | 1.02 (1.00, 1.04) | 1.02 (1.00, 1.04) |
|  |  |  |  |  |
| ^a^ Adjusted for age as time scale, year of first employment, smoking status and education (Model 1). Missing: tobacco smoking (n=76), education (n=20). Missing values were imputed in the multiple imputation analysis. | | | | |
| ^b^ Cox regression adapted to a case cohort design. | | | | |
| ^d^ Categorized into non-exposed (0) and tertiles among exposed. To capture the tail of the distribution, the upper tertile was divided into two by is median. | | | | |
| ^e^ Modelled by using the median of each exposure category to test for linear trend. | | | | |
| ^f^ The benzene exposure metrics (average intensity and cumulative) were based on an intensity score derived from a task-based semi-quantitative JEM. | | | | |

### Table S6:

| Hazard ratios (HR) of first primary bladder cancer and 95% confidence interval (95% CI) according to lagged benzene exposures in the Norwegian Offshore Petroleum Workers cohort followed 1999–2017. | | | | |
| --- | --- | --- | --- | --- |
|  | **Complete-case analyses** ^a^  **(n=2,137)** | | | **Multiple imputation analyses** ^a^ **(n=2,231)** ^b^ |
| **Benzene metric** | **No. of cases** | **Person-Years** | **HR (95% CI)** ^c^ | **HR (95% CI)** ^c^ |
| **Duration (years)** ^d^ |  |  |  |  |
| 10-Year Lag ^e, f^ |  |  |  |  |
| 0 | 38 | 10241.00 | 1.00 (reference) | 1.00 (reference) |
| >0–<5.5 | 41 | 8309.62 | 1.33 (0.83, 2.12) | 1.29 (0.82, 2.04) |
| 5.5–<13.3 | 36 | 7507.60 | 1.31 (0.82, 2.11) | 1.22 (0.76, 1.95) |
| 13.3–<18.8 | 20 | 3828.24 | 1.44 (0.80, 2.60) | 1.45 (0.81, 2.58) |
| 18.8–33.5 | 18 | 2342.85 | 1.80 (0.97, 3.34) | 1.93 (1.08, 3.47) |
| P-trend ^g^ |  |  | 0.068 | 0.057 |
| Continuous | 153 | 32229.32 | 1.02 (0.99, 1.04) | 1.02 (1.00, 1.04) |
|  |  |  |  |  |
| 15-Year Lag ^e, f^ |  |  |  |  |
| 0 | 40 | 11513.02 | 1.00 (reference) | 1.00 (reference) |
| >0–<5.5 | 45 | 8900.32 | 1.42 (0.91, 2.21) | 1.37 (0.89, 2.12) |
| 5.5–<13.3 | 40 | 7606.75 | 1.49 (0.94, 2.36) | 1.42 (0.90, 2.23) |
| 13.3–<18.8 | 15 | 2837.37 | 1.22 (0.65, 2.32) | 1.31 (0.71, 2.40) |
| 18.8–33.5 | 13 | 1371.86 | 1.76 (0.88, 3.49) | 1.71 (0.88, 3.31) |
| P-trend ^g^ |  |  | 0.107 | 0.152 |
| Continuous | 153 | 32229.32 | 1.02 (0.99, 1.04) | 1.02 (0.99, 1.04) |
|  |  |  |  |  |
| 20-Year Lag ^e, f^ |  |  |  |  |
| 0 | 48 | 14144.36 | 1.00 (reference) | 1.00 (reference) |
| >0–<5.5 | 47 | 9769.12 | 1.38 (0.90, 2.10) | 1.33 (0.88, 2.01) |
| 5.5–<13.3 | 38 | 6059.52 | 1.57 (0.98, 2.53) | 1.49 (0.93, 2.38) |
| 13.3–<18.8 | 15 | 1753.75 | 1.65 (0.87, 3.10) | 1.71 (0.94, 3.12) |
| 18.8–33.5 | 5 | 502.58 | 1.52 (0.56, 4.17) | 1.62 (0.64, 4.12) |
| P-trend ^g^ |  |  | 0.051 | 0.059 |
| Continuous | 153 | 32229.32 | 1.02 (0.99, 1.05) | 1.02 (0.99, 1.05) |
|  |  |  |  |  |
| **Cumulative benzene exposure** ^d, h^ |  |  |  |  |
| 10-Year Lag ^e, f^ |  |  |  |  |
| 0 | 38 | 10241.0 | 1.00 (reference) | 1.00 (reference) |
| >0–<2.0 | 40 | 7939.68 | 1.32 (0.83, 2.10) | 1.26 (0.80, 2.00) |
| 2.0–<7.6 | 35 | 7378.62 | 1.32 (0.81, 2.12) | 1.29 (0.81, 2.06) |
| 7.6–<15.3 | 18 | 3545.78 | 1.37 (0.74, 2.51) | 1.34 (0.74, 2.43) |
| 15.3–51.4 | 22 | 3124.24 | 1.79 (1.01, 3.16) | 1.79 (1.03, 3.12) |
| P-trend ^g^ |  |  | 0.084 | 0.065 |
| Continuous | 153 | 32229.32 | 1.02 (1.00, 1.04) | 1.02 (1.00, 1.04) |
|  |  |  |  |  |
| 15-Year Lag ^e, f^ |  |  |  |  |
| 0 | 40 | 11513.02 | 1.00 (reference) | 1.00 (reference) |
| >0–<2.0 | 43 | 8230.60 | 1.40 (0.90, 2.18) | 1.34 (0.86, 2.08) |
| 2.0–<7.6 | 35 | 7074.06 | 1.37 (0.85, 2.21) | 1.35 (0.85, 2.14) |
| 7.6–<15.3 | 14 | 3099.66 | 1.17 (0.60, 2.26) | 1.16 (0.61, 2.20) |
| 15.3–51.4 | 21 | 2311.98 | 2.07 (1.16, 3.70) | 2.07 (1.18, 3.64) |
| P-trend ^g^ |  |  | 0.059 | 0.045 |
| Continuous | 153 | 32229.32 | 1.02 (1.00, 1.04) | 1.02 (1.00, 1.04) |
|  |  |  |  |  |
| 20-Year Lag ^e, f^ |  |  |  |  |
| 0 | 48 | 14144.36 | 1.00 (reference) | 1.00 (reference) |
| >0–<2.0 | 43 | 8396.90 | 1.38 (0.89, 2.12) | 1.35 (0.88, 2.05) |
| 2.0–<7.6 | 31 | 6119.52 | 1.34 (0.82, 2.20) | 1.31 (0.81, 2.12) |
| 7.6–<15.3 | 17 | 2253.08 | 1.74 (0.92, 3.26) | 1.60 (0.86, 2.99) |
| 15.3–51.4 | 14 | 1315.46 | 2.15 (1.12, 4.13) | 2.16 (1.15, 4.06) |
| P-trend ^g^ |  |  | 0.025 | 0.026 |
| Continuous | 153 | 32229.32 | 1.03 (1.00, 1.05) | 1.03 (1.00, 1.05) |
|  |  |  |  |  |
| ^a^ Adjusted for age as time scale, year of first employment, smoking status and education (Model 1). Missing: tobacco smoking (n=76), education (n=20). Missing values were imputed in the multiple imputation analysis. | | | | |
| ^c^ Cox regression adapted to a case cohort design. | | | | |
| ^b^ Study sample consisting of 2231 male workers, including 160 first primary bladder cancer cases and 2071 non-cases. | | | | |
| ^d^ Time-dependent exposure. | | | | |
| ^e^ Disregarding exposure during the most recent 10-, 15- or 20-year period before any time-point (t) during follow-up. | | | | |
| ^f^ Categorized into unexposed (0) and tertiles among exposed. To capture the tail of the distribution, the upper tertile was divided into two by its median. | | | | |
| ^g^ Modelled by using the median of each exposure category to test for linear trend. | | | | |
| ^h^ The cumulative benzene exposure metric was based on an intensity score derived from a task-based semi-quantitative JEM. | | | | |

**References**

1 Steinsvåg, K., Bråtveit, M. & Moen, B. E. Exposure to carcinogens for defined job categories in Norway’s offshore petroleum industry, 1970 to 2005. *Occup Environ Med* **64**, 250-258 (2007).

2 Steinsvåg, K., Bråtveit, M. & Moen, B. E. Eksponering for kreftfremkallende faktorer i norsk offshore petroleumsvirksomhet 1970–2005. [Exposure to carsinogenic agents in the Norwegian offshore petroleum industry 1970–2005]. *University of Bergen: Bergen, Norway* (2005). https://w2.uib.no/filearchive/eksponering-for-kreftfremkallende-faktorer-i-norsk-offshore-petroleumsvirksomhet1970-2005_1_1.pdf.
